# Iron mitigates DMT1-mediated manganese cytotoxicity via the ASK1-JNK signaling axis: Implications of iron supplementation for manganese toxicity

**DOI:** 10.1038/srep21113

**Published:** 2016-02-16

**Authors:** Yee Kit Tai, Katherine C. M. Chew, Bryce W. Q. Tan, Kah-Leong Lim, Tuck Wah Soong

**Affiliations:** 1 Department of Physiology, Yong Loo Lin School of Medicine, National University of Singapore, Singapore 117597; 2 NUS Graduate School for Integrative Science and Engineering, and Neurobiology/Ageing Programme, Singapore 117456; 3 National Neuroscience Institute, Singapore 308433; 4 Duke-NUS Graduate Medical School, Singapore 169857

## Abstract

Manganese (Mn^2+^) neurotoxicity from occupational exposure is well documented to result in a Parkinson-like syndrome. Although the understanding of Mn^2+^ cytotoxicity is still incomplete, both Mn^2+^ and Fe^2+^ can be transported via the divalent metal transporter 1 (DMT1), suggesting that competitive uptake might disrupt Fe^2+^ homeostasis. Here, we found that DMT1 overexpression significantly enhanced Mn^2+^ cytoplasmic accumulation and JNK phosphorylation, leading to a reduction in cell viability. Although a robust activation of autophagy was observed alongside these changes, it did not trigger autophagic cell death, but was instead shown to be essential for the degradation of ferritin, which normally sequesters labile Fe^2+^. Inhibition of ferritin degradation through the neutralization of lysosomal pH resulted in increased ferritin and enhanced cytoplasmic Fe^2+^ depletion. Similarly, direct Fe^2+^ chelation also resulted in aggravated Mn^2+^-mediated JNK phosphorylation, while Fe^2+^ repletion protected cells, and this occurs via the ASK1-thioredoxin pathway. Taken together, our study presents the novel findings that Mn^2+^ cytotoxicity involves the depletion of the cytoplasmic Fe^2+^ pool, and the increase in autophagy-lysosome activity is important to maintain Fe^2+^ homeostasis. Thus, Fe^2+^ supplementation could have potential applications in the prevention and treatment of Mn^2+^-mediated toxicity.

Although manganese (Mn^2+^) is a trace metal element vital for biological functions, chronic exposure to Mn^2+^ has been associated with the development of neurological dysfunction resembling Parkinson’s disease (PD)^1^. Airborne Mn^2+^ exposure in humans is a cause of neurotoxicity to the basal ganglia, resulting in a mixture of neuropsychiatric and motoric disturbances known as manganism^2^. Chronic Mn^2+^ exposure is associated with a greater risk of developing PD amongst miners and welders, as well as populations living near ferroalloy industries^3^. In addition, the use of methylcyclopentadienyl manganese tricarbonyl (MMT) as a gasoline additive in some parts of the world could pose a danger to public health as MMT has been shown to be toxic to dopaminergic neurons^4^. With its extensive use in various industries, Mn^2+^ exposure could be a silent pandemic affecting neuronal development, as well as the onset and course of neurodegeneration^5^. The proposed mechanism of Mn^2+^ uptake into the brain involves the divalent metal transporter 1 (DMT1), a 12-transmembrane domain protein found in a range of tissues including duodenum, kidney and brain, and capable of transporting a number of divalent cations^6^. As both Mn^2+^ and Fe^2+^ rely heavily on DMT1 for cellular transport, prolonged exposure or chronic deficiency of either metal ion may interfere with the uptake and therefore the normal function of the other^7^. Indeed, Mn^2+^ levels in humans and animals have been observed to be influenced by Fe^2+^ status, specifically Mn^2+^ loading during Fe^2+^ deficiency^7^,^8^,^9^,^10^,^11^. While epidemiological and animal studies have demonstrated the detrimental effects of Mn^2+^exposure, the cell signaling pathways involved in Mn^2+^ toxicity and its interaction with Fe^2+^ are still not well understood. In our current study, we demonstrate that Mn^2+^ mediates cytotoxicity via depletion of cytoplasmic Fe^2+^ and activation of the JNK pathway. Importantly, we show that Fe^2+^-repletion suppressed Mn^2+^-mediated JNK activation, suggesting that Fe^2+^ supplementation can modify Mn^2+^-mediated cytotoxicity. Furthermore, our findings implicate the importance of the autophagic-lysosomal pathway in the degradation of ferritin since lysosomal inhibition further exacerbated cellular stress. Finally, we reduced JNK activation by overexpressing thioredoxin protein or inhibiting ASK1, suggesting the involvement of ASK1 upstream of the JNK pathway in Mn^2+^ toxicity. Taken together, our results provide evidence that Mn^2+^ cytotoxicity is related to Fe^2+^ depletion and the activation of JNK signaling via the thioredoxin/ASK1 pathway mediates cell death. These findings have important implications for the use of Fe^2+^ supplementation to reduce Mn^2+^ loading and toxicity in high risk populations.

## Results

### DMT1 overexpression in neuronal SH-SY5Y cells

**Figure 1:**
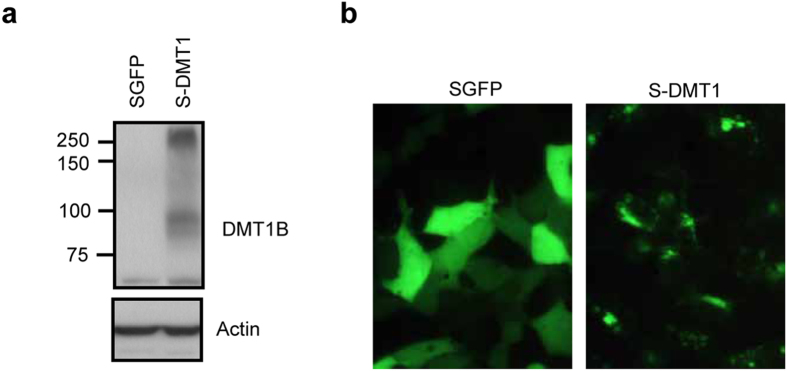
DMT1 overexpression in SH-SY5Y cells. (**a**) Representative western blot of DMT1 overexpression in S-DMT1 stable cell line using monoclonal DMT1B antibody. (**b**) Fluorescent images of vector and DMT1 stable cell lines showing differential GFP distribution (40X objective lenses).

To test the hypothesis that DMT1 mediates Mn^2+^ cytotoxicity, we used a previously generated geneticin-resistant stable SH-SY5Y cell line overexpressing DMT1 (S-DMT1)^12^ to transport Mn^2+^ into the cell. The expression of the DMT1-GFP fusion protein was verified using western blot analysis and fluorescence microscopy. DMT1-GFP protein is highly expressed in the S-DMT1 cells, as detected using DMT1B monoclonal antibody (Fig. 1A). The vector cell line overexpressing GFP protein (SGFP) showed the expression of the endogenous DMT1 at approximately 66 kDa while S-DMT1 cells showed further DMT1 immunoreactivity at 90 kDa and 250 kDa. The 90 kDa and 250 kDa bands correspond to DMT1-GFP and glycosylated DMT1-GFP fusion proteins^13^ respectively. Additionally, the DMT1-GFP protein was also viewed directly using fluorescence imaging to validate the cellular distribution of GFP compared to DMT1-GFP (Fig. 1B). In the S-DMT1 cells, DMT1-GFP was observed as puncta and located at the periphery of the cell, while SGFP cells showed diffuse cytoplasmic GFP expression. Indeed, studies of DMT1 transfected into non-neuronal cells such as Hep2 and CHO cells also confirm its plasma membrane and lysosomal localization^14^,^15^. Together, our data demonstrates that the localization of DMT1 overexpressed in SH-SY5Y cells is consistent with that of endogenous DMT1 in primary neuronal cells^16^.

### DMT1-mediated Mn^2+^ uptake, cytoplasmic accumulation and reduction in cell viability

**Figure 2:**
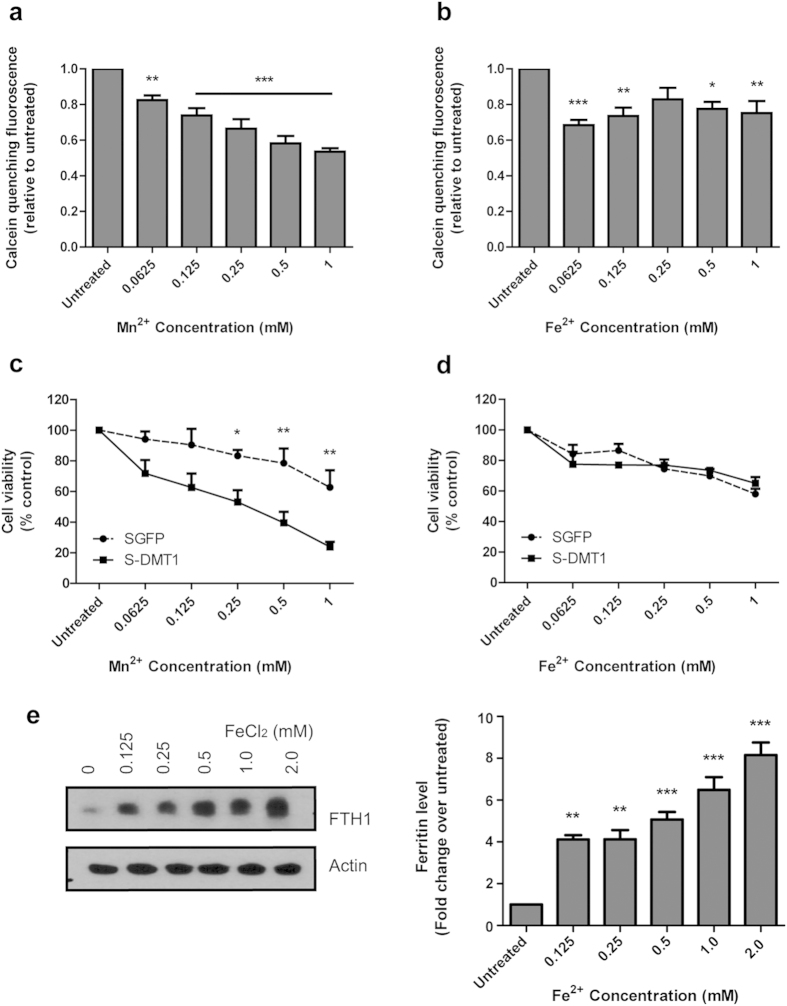
Uptake and cytoplasmic accumulation of Mn^2+^. Calcein quenching assay was performed on S-DMT1 cells treated with either Mn^2+^ or Fe^2+^ for 30 min. The effect of cytoplasmic accumulation on cell viability was determined using MTT assay. (**a**) Mn^2+^ treatment reduced calcein fluorescence in a concentration-dependent manner (compared to untreated, **p < 0.01; ***p < 0.001, n = 4, one-way ANOVA, Dunnett post hoc test). (**b**) Fe^2+^ treatment reduced calcein fluorescence (compared to untreated, *p < 0.05, **p < 0.01, ***p < 0.001, n = 4) but not in a concentration-dependent manner. (**c**) MTT assay of Mn^2+^ treatment of S-DMT1 cells for 24 h reduced cell viability compared to vector cells, especially for 0.5 and 1 mM Mn^2+^ (*p < 0.05, **p < 0.01, n = 4, two-way ANOVA, Bonferroni post hoc test). (**d**) MTT assay of Fe^2+^ treatment of S-DMT1 cells for 24 h did not show any significant reduction in cell viability compared to vector cells (n = 4). (**e**) Representative western blot showing upregulation of ferritin heavy chain (FTH1) protein in a Fe^2+^ concentration-dependent fashion in S-DMT1 cells treated for 12 h. Corresponding bar chart displays ferritin protein levels with Fe^2+^ treatment (**p < 0.01, ***p < 0.001, n = 3), with error bars representing standard error of mean (S.E.M.). *β*-actin was used as a loading control.

Previously, we have shown that the overexpression of DMT1 mediates robust cellular entry of Fe^2+^ into SH-SY5Y cells using the Fe^55^ uptake assay 12. As this assay measures the absolute accumulation of Fe^2+^, the difference between intracellular labile and non-labile Fe^2+^ could not be distinguished. To examine the accumulation of chelatable labile Fe^2+^ and Mn^2+^, cell-permeant Calcein-AM (a fluorescent dye whose intensity can be quenched by metal ions) was used. Calcein-AM is known to permeate the plasma membrane but does not readily enter subcellular organelles. This allows us to estimate the entry and cytoplasmic retention of labile Mn^2+^ and Fe^2+^ by measuring the amount of calcein fluorescence that is being quenched by the metal ions^17^. When S-DMT1 cells were treated with Mn^2+^ for 30 min, there was a concentration-dependent quenching of calcein fluorescence (Fig. 2A). In contrast, while Fe^2+^ treatment mediated a reduction in calcein fluorescence, the reduction itself was not concentration-dependent (Fig. 2B). This indicates that DMT1 mediates robust entry and accumulation of labile Mn^2+^. In parallel with this accumulation, S-DMT1 cells treated with Mn^2+^ for 24 h displayed significant reduction in cell viability compared to vector SGFP cells (Fig. 2C). Unlike Mn^2+^ however, increasing concentrations of Fe^2+^ were not associated with the progressive accumulation of its labile form. Moreover, in contrast to Mn^2+^, Fe^2+^ treatment did not result in any significant reduction in S-DMT1 cell viability compared to SGFP cells (Fig. 2D). This suggests that cells may have a protective mechanism against the build-up of labile iron. A likely candidate in this case would be the ferritin heavy chain (FTH1), which converts Fe^2+^ into the non-labile Fe^3+^ form. Indeed, we found that the level of ferritin was dramatically upregulated in cells treated with Fe^2+^ for 12 h and this occurred in a concentration-dependent fashion (Fig. 2E).

### Cell viability reduction mediated by Mn^2+^ is associated with the activation of MAP Kinase JNK pathway

**Figure 3:**
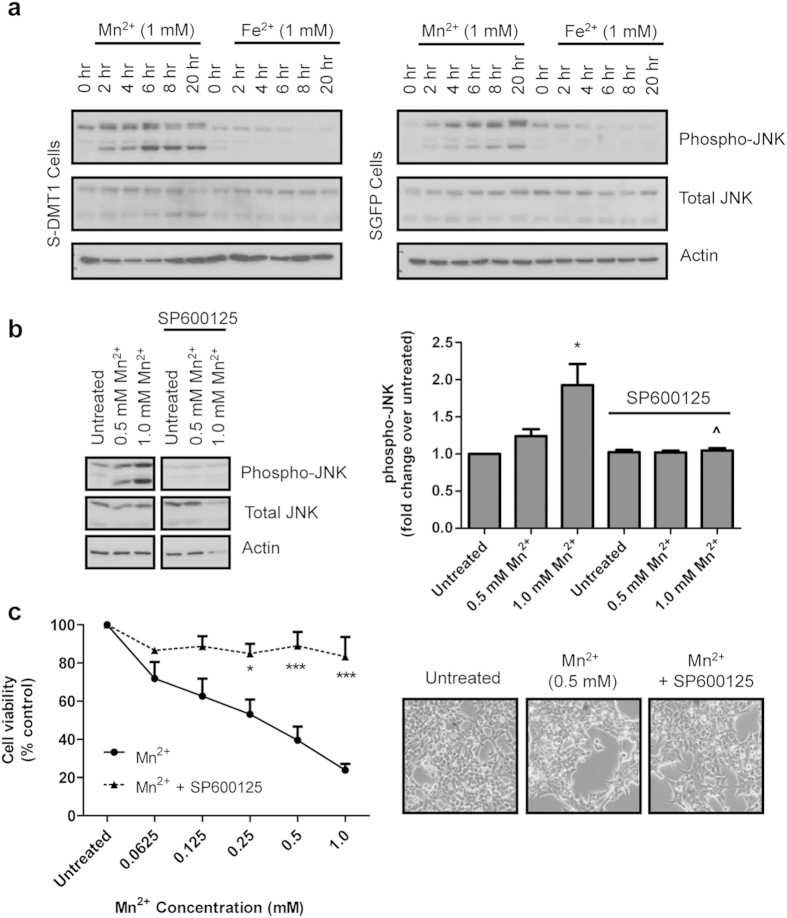
Mn^2+^ cytotoxicity is associated with JNK activation. (**a**) Representative western blot showing time-dependent JNK phosphorylation upon treatment with 1 mM of Mn^2+^ in both S-DMT1 and vector cells. S-DMT1 cells displayed more robust JNK phosphorylation compared to vector cells. 1 mM Fe^2+^ treatment of both cell lines did not increase JNK phosphorylation. (**b**) S-DMT1 cells treated with 1 mM of Mn^2+^ for 12 h increased (*p < 0.05) JNK phosphorylation compared to untreated. This JNK phosphorylation induced by Mn^2+^ was significantly (^*p* < 0.05) reduced with SP600125 (25 μM) treatment. Bar chart shows fold change of phosphorylated JNK protein levels over untreated (n = 3, Student’s *t*-test), with error bars representing standard error of mean (S.E.M.). Corresponding full-length blots are presented in Supplementary Fig. 1. (**c**) MTT cell viability assay showing the protective effect of SP600125 against Mn^2+^-mediated cytotoxicity (*p < 0.05, ***p < 0.001, n = 3, two-way ANOVA, Bonferroni post hoc test). Phase contrast images show the reduced vulnerability of S-DMT1 cells to 0.5 mM Mn^2+^ cytotoxicity when treated with SP600125 for 24 h.

Given that the MAP kinase stress-induced pathway plays an important role in the regulation of cell proliferation, differentiation, survival and apoptosis^18^, we next sought to examine the potential effect of Mn^2+^ on this pathway. At 1 mM Mn^2+^, S-DMT1 cells showed a dramatic increase in JNK phosphorylation in a time-dependent fashion compared to SGFP cells (Fig. 3A, blots were developed with the same exposure time). Treatment with 1 mM Fe^2+^ did not significantly increase JNK phosphorylation in both SGFP and S-DMT1 cells. Since Mn^2+^ treatment of S-DMT1 cells resulted in enhanced JNK phosphorylation, we next questioned whether JNK phosphorylation was responsible for Mn^2+^-mediated reduction in cell viability. To examine this, we used 25 μM of SP600125, an inhibitor of JNK phosphorylation. S-DMT1 cells treated with 0.5 or 1 mM Mn^2+^ for 12 h showed increased JNK phosphorylation compared to untreated cells. This phosphorylation was significantly suppressed with addition of SP600125 (Fig. 3B). Notably, the more than 2-fold increase in JNK phosphorylation mediated by 1 mM Mn^2+^ treatment was totally abolished in the presence of SP600125. Under phase contrast microscopy, S-DMT1 cells treated with SP600125 showed reduced vulnerability to Mn^2+^ compared to cells treated with Mn^2+^ alone. Indeed, using the MTT cell viability assay, S-DMT1 cells treated with a range of Mn^2+^ concentrations for 24 h were significantly protected against Mn^2+^ cytotoxicity in the presence of SP600125 (Fig. 3C). The data suggests that JNK activation plays an important role in Mn^2+^-mediated cell death.

### Autophagic activity associated with Mn^2+^ treatment is protective during Mn^2+^ stress

**Figure 4:**
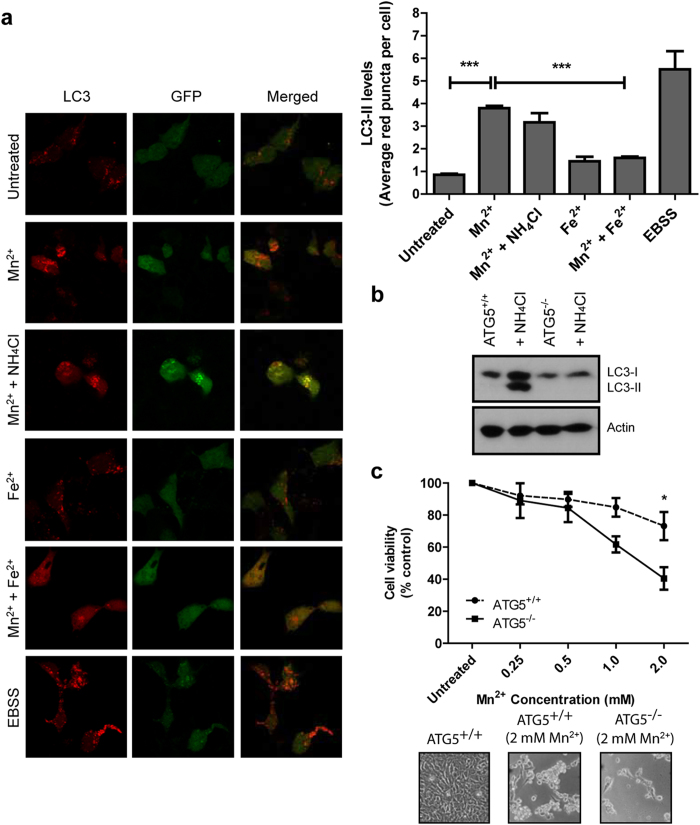
Mn^2+^-mediates an increase in autophagy, which is protective during Mn^2+^ stress. (**a**) SH-SY5Y cells were transfected with tandem fluorescence LC3 containing GFP and RFP for 24 h. Cells treated with 0.5 mM Mn^2+^ for 6 h had a significantly increased (***p < 0.001) number of red LC3 puncta per cell (at least 50 cells per experiment). 0.5 mM Fe^2+^ treatment did not show any significant increase in LC3 puncta and co-treatment of 0.5 mM Fe^2+^ and 0.5 mM Mn^2+^ substantially (***p < 0.001) reduced LC3 puncta compared to Mn^2+^ alone. Treatment with NH_4_Cl neutralized lysosome pH, allowing the appearance of yellow LC3 puncta indicating autophagic flux. EBSS was used as a positive control for autophagy induction. Bar chart shows the quantification of average LC3 red puncta per cell for various treatments (n = 3, Student’s *t*-test). (**b**) Representative western blot of ATG5^+/+^ and ATG5^−/−^ MEF with or without NH_4_Cl treatment. NH_4_Cl inhibited autophagy degradation, allowing the accumulation and visualization of the LC3-II protein in normal ATG5^+/+^ MEF but not ATG5^−/−^. (**c**) MTT assay of ATG5^+/+^ and ATG5^−/−^ MEF treated with Mn^2+^ for 24 h showing the significant reduction (*p < 0.05, n = 3, two-way ANOVA, Bonferroni post hoc test) in ATG5^−/−^ cell viability at 2 mM Mn^2+^ compared to ATG5^+/+^ MEF. Corresponding phase contrast images of 2 mM Mn^2+^ treatment for 24 h showed similar increased vulnerability of ATG5^−/−^ MEF.

In our previous work, we showed that chronic Fe^2+^ overload together with α-synuclein A53T overexpression led to an increase in autophagic activity and cell death^12^. We thus investigated whether autophagy could be involved in Mn^2+^-mediated cytotoxicity. Upon autophagy induction, cytoplasmic LC3-I is lipidated to LC3-II, which then localizes to autophagic vesicles^19^. To determine the level of autophagic activity in Mn^2+^-treated cells, naïve SH-SY5Y cells were transfected with the mRFP-GFP tandem fluorescence-tagged LC3 construct (tf-LC3) for 24 h. Transfected cells treated with 0.5 mM Mn^2+^ for 6 h showed induction of autophagy as indicated by the increase in average red LC3 puncta per cell compared to untreated cells (Fig. 4A). The green GFP puncta associated with tf-LC3 was lost in Mn^2+^-treated cells presumably due to the quenching of the GFP signal within the acidic lysosomes. To reverse the loss of GFP puncta signal, cells were co-treated with Mn^2+^ and NH_4_Cl (which neutralizes the pH of lysosomes) and the GFP signal reappeared and colocalized with the red LC3 puncta in the merged image. Cells treated with either 0.5 mM Fe^2+^ or 0.5 mM Fe^2+^ + Mn^2+^ for 6 h did not show any significant increase in the number of LC3 puncta, suggesting that autophagy was not significantly changed with these treatments. Cells treated with EBSS for 6 h (which promotes starvation-induced autophagy) was used as a positive control for autophagy induction. In addition, we used western blot analysis to detect the level of LC3-II, which acts as a marker for autophagy. S-DMT1 cells treated with 0.5 mM Mn^2+^ for 12 h showed a substantial accumulation of LC3-II compared to untreated cells (Supplementary Fig. 2A), while 0.5 mM Fe^2+^ and 0.5 mM Fe^2+^ + Mn^2+^ treatments did not result in any significant change. The data suggests that Mn^2+^ mediates robust autophagy activation in S-DMT1 cells, while this was not observed with Fe^2+^. Importantly, by simply co-treating with Fe^2+^, the activation of autophagy induced by Mn^2+^ could be reversed.

With the robust activation of autophagy upon Mn^2+^ stress, we examined the effect of autophagy on cell viability by using ATG5 autophagy-deficient mouse embryonic fibroblast cells (ATG5^−/−^ MEF). ATG5 is an important gene required for the initiation of autophagy^20^,^21^. Treatment of ATG5^+/+^ and ATG5^−/−^ MEF with NH_4_Cl for 12 h neutralized lysosome pH, preventing autophagic degradation and allowing the accumulation of LC3-II protein in the ATG5^+/+^ MEF but not in ATG5^−/−^ (Fig. 4B). This verifies the failure of ATG5^−/−^ MEF to initiate autophagy. We then carried out the MTT cell viability assay and found that ATG5^−/−^ MEF treated with 2 mM Mn^2+^ showed significant reduction in cell viability compared to ATG5^+/+^ MEF. Under phase contrast microscopy, autophagy deficient ATG5^−/−^ MEF also showed increased vulnerability to Mn^2+^ cytotoxicity (Fig. 4C). As lysosomal acidification is important for proteolysis of ferritin to extract labile Fe^2+^ 22, the inhibition of lysosomal function by NH_4_Cl could potentially aggravate Mn^2+^-mediated cytotoxicity. To test this, we treated S-DMT1 cells with NH_4_Cl and found that Mn^2+^-mediated JNK phosphorylation was indeed significantly higher from 2 h onwards compared to Mn^2+^ alone (Supplementary Fig. 2B). In addition the inhibition of lysosomal function could be seen from the accumulation of autophagic LC3-II, which would otherwise be degraded by the lysosomes.

### Mn^2+^ induces ferritin degradation and Fe^2+^ chelation exacerbates Mn^2+^-mediated JNK activation

**Figure 5:**
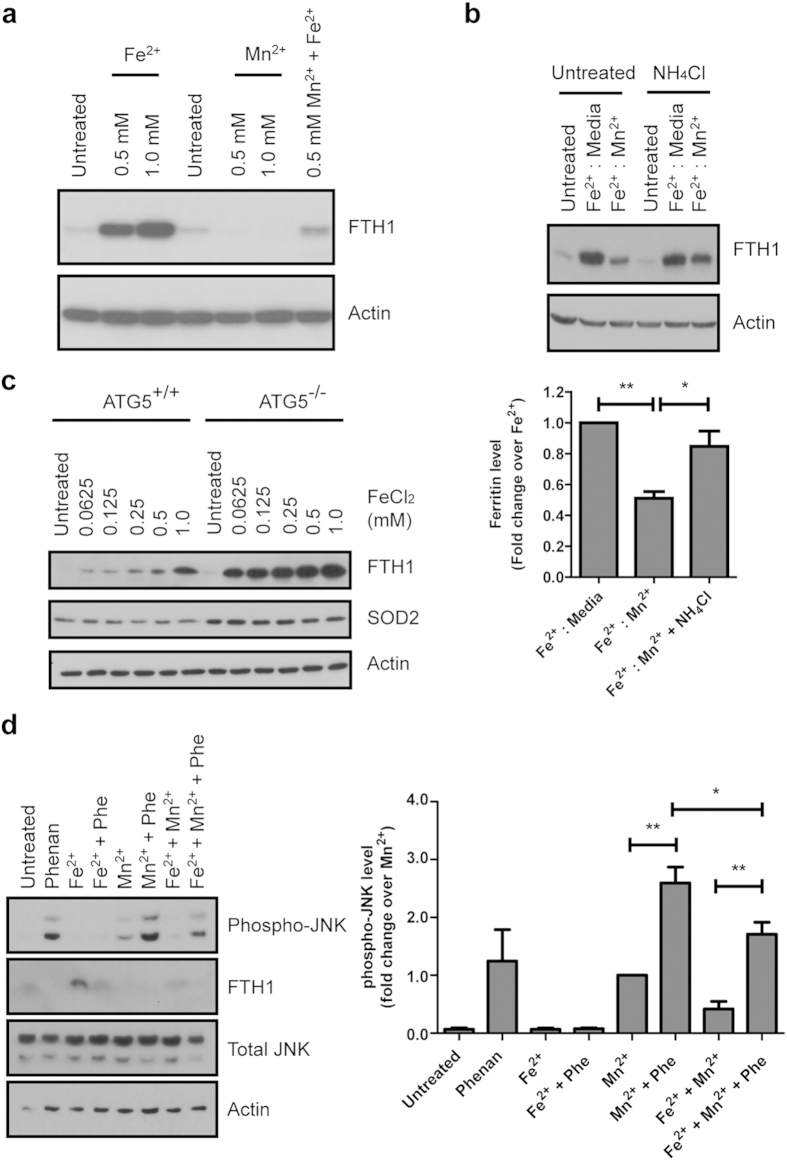
Mn^2+^ induces ferritin degradation and Fe^2+^ chelation exacerbates Mn^2+^-mediated JNK activation. (**a**) Representative western analysis of ferritin levels upon Fe^2+^ and Mn^2+^ treatment. 0.5 and 1 mM of Fe^2+^ for 24 h resulted in a concentration-dependent increase in ferritin levels in S-DMT1 cells. In contrast, both 0.5 and 1 mM Mn^2+^ reduced ferritin levels compared to untreated. Ferritin loss associated with 0.5 mM Mn^2+^ was partially reversed when co-treated with 0.5 mM Fe^2+^. (**b**) Representative western analysis of S-DMT1 cells pretreated with 0.5 mM Fe^2+^ for 12 h (to upregulate ferritin) before treatment with 0.5 mM Mn^2+^ for another 12 h, with or without autophagy inhibition. In Fe^2+^ pretreated cells, incubation with Mn^2+^ significantly (**p < 0.01) reduced ferritin levels compared to Fe^2+^ treatment alone. With NH_4_Cl, Mn^2+^-induced reduction of ferritin is significantly suppressed (*p < 0.05) compared to Mn^2+^ without inhibitor. Corresponding bar chart shows ferritin protein fold change over Fe^2+^ treatment (n = 3, Student’s *t*-test), with error bars representing standard error of mean (S.E.M.). (**c**) Increasing concentration of Fe^2+^ led to marked ferritin accumulation in autophagy deficient ATG5^−/−^ MEF compared to ATG5^+/+^. Autophagy deficiency in the ATG5^−/−^ MEF was verified through the increase in mitochondrial SOD2 compared to ATG5^+/+^ MEF. (**d**) Mn^2+^ treatment together with Fe^2+^ chelation by 1,10-phenanthroline for 12 h resulted in increased JNK phosphorylation compared to Mn^2+^ alone (**p < 0.01, n = 4, Student’s *t*-test). In contrast, phenanthroline in the presence of Fe^2+^ did not result in increased JNK phosphorylation due to sufficient Fe^2+^. Similarly, addition of Fe^2+^ to Mn^2+^/phenanthroline reduced JNK phosphorylation (*p < 0.05).

Since addition of Fe^2+^ appeared to reduce the level of autophagy induced by Mn^2+^ (Fig. 4A), we next investigated the role of Mn^2+^-mediated autophagy with regards to Fe^2+^ homeostasis. We found that S-DMT1 cells treated with 0.5 or 1 mM Mn^2+^ for 24 h showed a significant reduction in ferritin protein compared to untreated (Fig. 5A). In contrast, treatment with 0.5 or 1 mM Fe^2+^ resulted in a concentration-dependent upregulation of ferritin. Importantly, co-treatment of 0.5 mM Mn^2+^ and 0.5 mM Fe^2+^ prevented the complete loss of ferritin compared to 0.5 mM Mn^2+^ treatment alone. This led us to hypothesize that Mn^2+^ treatment resulted in enhanced degradation of ferritin due to cellular Fe^2+^ depletion. We then proceeded to determine if autophagy was involved in Mn^2+^-mediated ferritin loss. S-DMT1 cells were first exposed to Fe^2+^ for 12 h or left untreated. Subsequently, this was changed to normal media or 0.5 mM Mn^2+^ for another 12 h. Ferritin protein was dramatically reduced on treatment with Mn^2+^ (Fig. 5B). However, the addition of autophagy inhibitor NH_4_Cl almost completely prevented Mn^2+^-mediated degradation of ferritin.

Since lysosomal acidification is essential for the degradation of ferritin in order to release Fe^2+^ 23, we validated the importance of the basal autophagy-lysosomal pathway for the maintenance of Fe^2+^ homeostasis. Immunoblotting of ATG5^−/−^ MEF treated with increasing concentrations of Fe^2+^ for 12 h showed dramatic upregulation and accumulation of ferritin protein compared to ATG5^+/+^ MEF (Fig. 5C). In addition, mitochondrial SOD2 was observed to be relatively higher (independent of Fe^2+^ treatment) in the ATG5^−/−^ compared to ATG5^+/+^ MEF, indicating a deficiency in autophagic clearance of mitochondria these cells. This indicates that basal autophagy is indeed important to control ferritin degradation in response to changes in cellular Fe^2+^.

As Mn^2+^ treatment interferes with Fe^2+^ homeostasis by increasing autophagic clearance of ferritin, we asked whether enhancement of iron stores via Fe^2+^ repletion could abrogate Mn^2+^-associated JNK phosphorylation. S-DMT1 cells pre-exposed to 0.5 mM Fe^2+^ for 12 h showed increase in ferritin protein, while JNK phosphorylation remained unchanged (Supplementary Fig. 3A). In contrast, Mn^2+^ treatment increased JNK phosphorylation and reduced ferritin level. Finally, Fe^2+^-repletion of Mn^2+^ treated cells reduced JNK phosphorylation and increased ferritin protein compared to Mn^2+^ treatment alone. Since treatment with Fe^2+^ could reduce Mn^2+^-mediated JNK phosphorylation, we next examined the viability of S-DMT1 cells using the MTT assay. Indeed, cells treated with both Mn^2+^ and Fe^2+^ were significantly protected against Mn^2+^ cytotoxicity (Supplementary Fig. 3B). Additionally, the cells appeared healthier as observed using phase contrast microscopy.

We next examined the response of S-DMT1 cells to the reverse experiment by inducing Fe^2+^ deficiency with the cell permeable Fe^2+^ chelator 1,10-phenanthroline. In contrast to Fe^2+^ repletion, Fe^2+^ depletion resulted in significant toxicity. Treatment with 75 μM phenanthroline (Phen) alone for 12 h led to JNK phosphorylation, which could be reversed by supplementation with 0.5 mM Fe^2+^ (Fig. 5D). More importantly, Phen treatment exacerbated Mn^2+^ toxicity, resulting in a 2.6-fold increase in JNK phosphorylation. This, in turn, could be reduced by co-treatment with Fe^2+^, leading to a reduction of JNK phosphorylation for Fe^2+^/Mn^2+^/Phen compared to Mn^2+^/Phen. The results demonstrate the toxic effect of Fe^2+^ depletion and that enhancement of iron stores as well as extracellular competition between Fe^2+^ and Mn^2+^ for entry into cell via DMT1 could rescue Mn^2+^-mediated cytotoxicity.

### Thioredoxin overexpression and ASK1 inhibition reverse JNK phosphorylation mediated by Mn^2+^ and lysosomal inhibition

**Figure 6:**
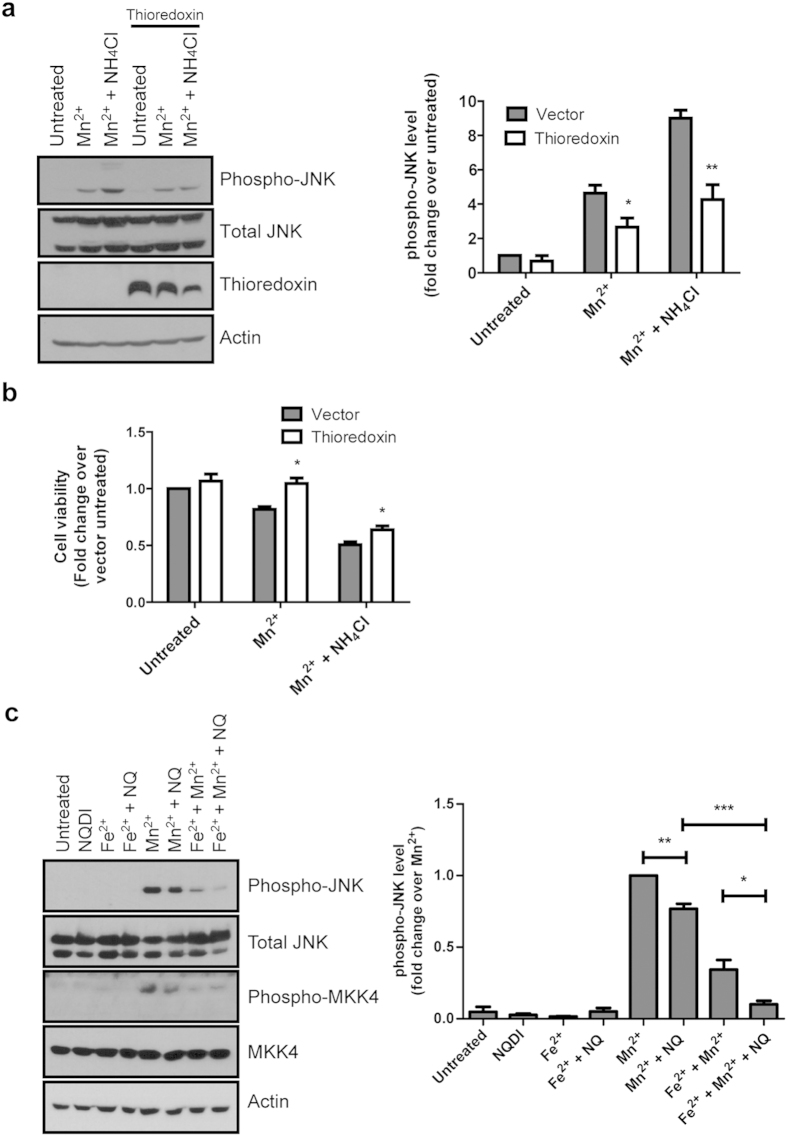
Thioredoxin overexpression and ASK1 inhibition reduce Mn^2+^-mediated JNK phosphorylation via the thioredoxin/ASK1/MKK4 pathway. (**a**) Representative western blot and bar chart showing the reduction in JNK phosphorylation mediated by thioredoxin transfection of Mn^2+^ and NH_4_Cl treated S-DMT1 cells (compared to vector transfected cells, *p < 0.05, **p < 0.01, n = 4, Student’s *t*-test). Error bars represent standard error of mean (S.E.M.). (**b**) MTT assay of thioredoxin transfected S-DMT1 cells treated for 24 h with Mn^2+^ or Mn^2+^ with NH_4_Cl (*p < 0.05, compared to corresponding vector transfected cells, n = 4). (**c**) Representative western blot and corresponding bar chart showing the reduction in MKK4 and JNK phosphorylation mediated by 12 h treatment with 10 μM ASK1 inhibitor NQDI-1 in S-DMT1 cells (*p < 0.05, **p < 0.01, ***p < 0.001, n = 4, Student’s *t*-test). *β*-actin was used as a control for equal loading.

**Figure 7:**
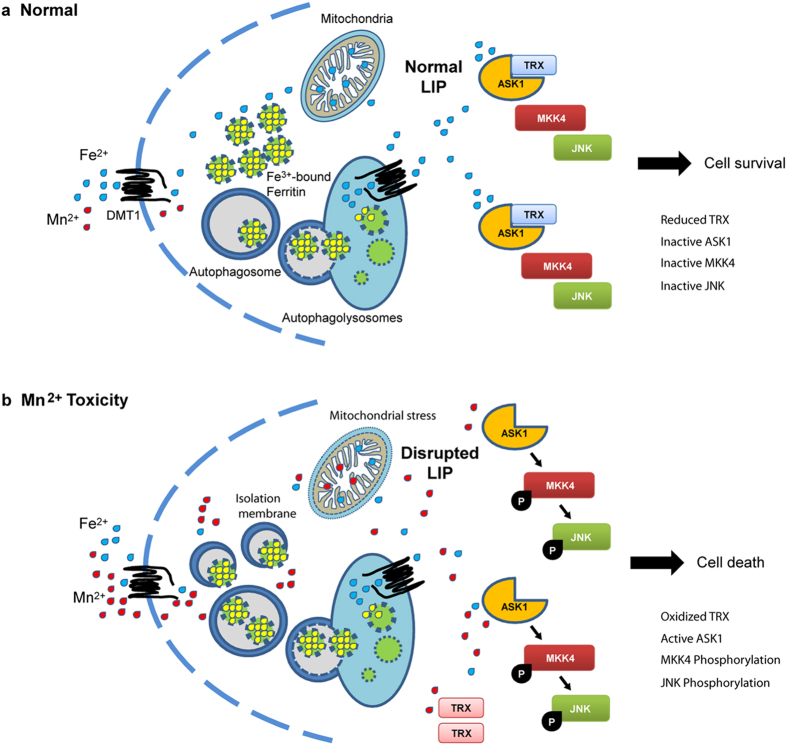
A proposed model of cellular Mn^2+^ toxicity via DMT1. (**a**) Under normal conditions, cells acquire adequate amounts of Fe^2+^ through cellular uptake via DMT1 and degradation of ferritin to maintain the labile iron pool (LIP). (**b**) However, Mn^2+^ overexposure competes with Fe^2+^ for uptake by DMT1. In addition, the accumulation Mn^2+^ in the cytoplasm disrupts LIP, resulting in enhanced autophagy to degrade ferritin for Fe^2+^ mobilization. Mn^2+^-mediated cellular iron depletion also results in the activation of JNK via the TRX-ASK1 pathway, leading to cell death.

Cellular Fe^2+^ depletion using Fe^2+^ chelators has been shown to stimulate the JNK pathway via the dissociation of the ASK1-thioredoxin complex, activation of ASK1 and the subsequent phosphorylation of JNK^24^. Hence, we sought to clarify the link between Mn^2+^-mediated JNK phosphorylation and cellular Fe^2+^ depletion by assessing the level of phosphorylated JNK in the presence of thioredoxin overexpression. S-DMT1 cells transfected with thioredoxin for 24 h showed a suppression of Mn^2+^-mediated JNK phosphorylation compared to vector-transfected cells. Additionally, thioredoxin overexpression reduced JNK phosphorylation induced by Mn^2+^ and NH_4_Cl (Fig. 6A). We then investigated whether thioredoxin overexpression could rescue cells from Mn^2+^ cytotoxicity. Mn^2+^ treatment of S-DMT1 cells transfected with thioredoxin for 24 h showed a significant increase in cell viability compared to Mn^2+^ treatment alone (Fig. 6B). Thioredoxin overexpression also increased cell viability for Mn^2+^ and NH_4_Cl treatment. Finally, we used NQDI-1, an ASK1 inhibitor^25^, to investigate the involvement of ASK1 and the downstream components MKK4 and JNK^26^. On treatment with Mn^2+^, MKK4 and JNK showed robust activation and this was reduced when NQDI-1 was added to inhibit ASK1 activity (Fig. 6C). Co-treatment of Mn^2+^ with Fe^2+^ also reduced MKK4 and JNK activation, presumably because Fe^2+^ supplementation reduced ASK1-thioredoxin dissociation and subsequent ASK1 activation. Taken together, this suggests that Mn^2+^ exposure results in cellular Fe^2+^ disruption with a concomitant increase in JNK phosphorylation via the thioredoxin/ASK1/MKK4 pathway (Fig. 7).

## Discussion

Nutritional iron deficiency affects a significant proportion of the world population. In 2011, it was estimated that around 800 million women and children suffer from anemia, of which 50% is attributed to iron-deficiency^27^. An important consequence of iron deficiency is an apparent heightened risk of heavy-metal poisoning, especially in children, which may affect their neurodevelopment. Iron-deficient individuals have an increased absorption capacity that is not specific to iron, resulting in increased absorption of other divalent heavy metals such as manganese, lead and cadmium^28^. Prevention of iron deficiency could thus reduce the number of children susceptible to heavy-metal poisoning from contaminated water, lead paint and pollution from automobile, industrial or welding fumes^27^.

Since the DMT1 transporter has been shown to mediate the uptake of both Mn^2+^ and Fe^2+^ in the brain^29^, we generated SH-SY5Y stable cell lines overexpressing DMT1 to better understand the signaling pathways involved in the interaction of these two ions. DMT1 facilitated a concentration-dependent uptake of Mn^2+^ into the cytoplasm. This cytoplasmic accumulation of labile Mn^2+^ contributed substantially to cell toxicity since there is no known storage protein for Mn^2+^. In contrast, cells treated with Fe^2+^ only showed a 25% increase in labile Fe^2+^, which did not significantly affect cell viability. This is due to the presence and upregulation of ferritin protein upon Fe^2+^ exposure. Ferritin is a Fe^3+^-storage protein with ferroxidase activity that protects cells against Fe^2+^-mediated cytotoxicity^30^.

Although Mn^2+^ and Fe^2+^ share similar physical and chemical properties, the cellular requirement for each metal ion is highly specific in order for them to catalyze reactions in their respective metabolic pathways. Consequently, in the event of cellular Mn^2+^ overexposure, the accumulation of this ion may interfere with the metabolic processes of Fe^2+^-requiring enzymes. With uptake by DMT1, the level of Mn^2+^ might reach a concentration high enough to interfere with the synthesis of Fe^2+^-requiring enzymes or even directly displace Fe^2+^. In our study, pre-treatment as well as co-treatment with Fe^2+^ rescued cells from Mn^2+^-mediated cytotoxicity. It is possible that co-treatment resulted in Fe^2+^ competing with Mn^2+^ for uptake via DMT1, reducing Mn^2+^ entry into the cell, while pre-treatment increased stores in ferritin that allowed subsequent Fe^2+^ release to counter the toxic effects of Mn^2+^. Thus, there appears to be a delicate balance of cellular Fe^2+^ which could be disrupted by Mn^2+^ overexposure (Fig. 7).

We also found that autophagy was essential for the degradation of iron-laden ferritin in order to maintain the necessary level of cellular Fe^2+^ during Mn^2+^ exposure. This was corroborated through the inhibition of lysosome function which prevented the degradation of ferritin and hence failure of Fe^2+^ release. Indeed, the role of autophagy in maintaining cellular Fe^2+^ balance is supported by the recent identification of the nuclear receptor coactivator 4 (NCOA4), which is responsible for the selective targeting of ferritin to the lysosomes for protein turnover^31^. NCOA4 co-localizes with ferritin and LC3B in autophagosomes upon upregulation of ferritin by ferric ammonium citrate. In addition, either ATG5 or NCOA4 knockdown through RNA interference retarded ferritin turnover. This provided a molecular explanation on how the bioavailability of Fe^2+^ is maintained within the cell through autophagy, a process which the authors referred to as ‘ferritinophagy’.

In summary, we elucidate for the first time the cellular mechanisms of how Mn^2+^ cytotoxicity is closely linked to the disruption of Fe^2+^ homeostasis. Mn^2+^ interferes with Fe^2+^ balance via entry through DMT1 as well as disruption to intracellular signaling mechanisms. Specifically, we found that autophagy is upregulated upon Mn^2+^ treatment and this does not lead to autophagic cell death, but instead plays a pro-survival role. Autophagy is paramount during Mn^2+^ stress in order to degrade ferritin, release labile Fe^2+^ and restore Fe^2+^ balance. Consequently, inhibition of the autophagy-lysosomal pathway and subsequent failure to degrade ferritin aggravated Mn^2+^ cellular stress. We also demonstrated that JNK signaling during Mn^2+^ cytotoxicity could be reduced with Fe^2+^ repletion, supporting the finding that Fe^2+^ depletion is involved during Mn^2+^ stress. These findings could be extended with further studies to determine the potential of Fe^2+^ supplementation to counter Mn^2+^ toxicity in populations at risk of chronic Mn^2+^ exposure.

## Methods

### Generation of DMT1-GFP and Vector Stable Cell Lines

cDNA of DMT1 (Accession: AF153279) without the iron-response element (IRE) at the 3′ UTR was subcloned into a GFP vector to generate DMT1-GFP. The generation and characterization of the DMT1 stable cell lines were performed by our laboratory and described previously^12^. The DMT1 stable cell line (S-DMT1) and empty vector control (SGFP) were maintained in 200 μg/ml G418 (Gibco) supplemented DMEM containing 10% FBS and 1% Pen/Strep in 5% CO_2_ at 37 °C. The TRX1-Flag plasmid was obtained from Addgene (plasmid: 21283) and was previously described^32^.

### ATG5^−/−^ Mouse Embryonic Fibroblast (MEF)

The autophagy-deficient immortalized MEF cells were kind gifts from Dr. Noboru Mizushima from Tokyo Medical and Dental University, via Dr Shen Han Ming, Department of Physiology, NUS. The MEF cells were maintained in DMEM containing 10% FBS and 1% Pen/Strep in 5% CO_2_ at 37 °C.

### Materials

Reagents were from Sigma Aldrich unless otherwise stated. NH_4_Cl (30 mM), and 1,10-Phenanthroline (75 μM) were dissolved in distilled water. SP600125 (25 μM), and NQDI-1 (10 μM, Cayman Chemical) were dissolved in DMSO. Mn^2+^ was purchased as a 1 M MgCl_2_ solution, while Fe^2+^ was prepared fresh by dissolving FeCl_2_ in 0.1 M HCl. Amino acid-free Earl’s balanced salt solution (EBSS) was used to induce autophagy via complete starvation.

### Cell transfection and treatment

Cell transfection was performed using Lipofectamine 2000 (Invitrogen) according to the manufacturer’s protocol. Cells of at least 70% confluency were transfected with the desired plasmid constructs for 24 h before being subjected to various treatments.

### Overexpression of LC3

The mRFP-GFP tandem fluorescent-tagged LC3 (tf-LC3) construct was previously described in our work^12^. Briefly, mRFP retained its red fluorescence in acidic lysosomes, but GFP fluorescence was quenched and could be recovered by neutralizing lysosome pH with NH_4_Cl. Transfection of tf-LC3 into SH-SY5Y cells was performed using Lipofectamine 2000 (Invitrogen). After 24 h, the cells were treated with various metal ions and chemical inhibitors for 6 h before fixing with 2.5% PFA and viewing with a confocal microscope (Olympus).

### Calcein-AM Metal Quenching Assay

Calcein acetoxymethylester (Calcein-AM, 0.25 μM) was dissolved in DMSO. Briefly, cells were treated with metal ions for 30 min, then rinsed with PBS and incubated with calcein-AM in DMEM for 20 min at 37 °C, 5% CO_2._ The cells were rinsed in PBS followed by complete lysis in RIPA buffer containing 150 mM NaCl, 1% Triton X-100, 0.5% sodium deoxycholate, 0.1% SDS and 50 mM Tris, pH 8. The supernatant was transferred to a 96-well plate for fluorescence analysis at 488/518 nm wavelength (Tecan Infinite 200 PRO multimode reader). The fluorescence of the lysates was normalized to protein concentration determined using BCA method (Thermo Scientific).

### Cell viability assay

Cell viability was determined using the MTT Cell Proliferation Kit (Roche) according to the manufacturer’s protocol. Cells were seeded into a 96-well plate to reach a confluency of 70%. The cells were then treated with Mn^2+^, Fe^2+^ or other chemicals for 24 h before analysis using MTT.

### Protein Lysis and Western Blot Analysis

Protein extraction was carried out by lysing cells in PBS buffer containing 1% Triton X-100 with added protease and phosphatase inhibitors (Roche). Protein content was determined using Bradford assay (Pierce). Approximately 25 to 70 μg of total protein was resolved on 8, 10 or 12% SDS-PAGE gel before transferring onto PVDF membrane (Millipore) for detection using ECL reagent (Pierce). The following antibodies were used: anti-phospho-JNK, anti-JNK, anti-phospho-MKK4, anti-MKK4, anti-FTH1 (Cell Signaling Technology), anti-TRX (Santa Cruz), anti-LC3B (Abgent), anti-SOD2 (Abcam), anti-actin and HRP-conjugated anti-rabbit and anti-mouse (Sigma).

### Densitometric Analysis and Statistics

Western blot band intensities were analyzed using the densitometric analysis software Quantity One (Biorad). The intensities of the specified bands were normalized to *β*-actin. Data was expressed as standard error of mean (S.E.M) of at least three independent experiments. Experimental data was compared using Student’s *t-*test, one-way ANOVA with Dunnett post hoc test or two-way ANOVA with Bonferroni post hoc test, and was considered statistically significant when *p* < 0.05.

## Additional Information

**How to cite this article**: Tai, Y. K. *et al.* Iron mitigates DMT1-mediated manganese cytotoxicity via the ASK1-JNK signaling axis: Implications of iron supplementation for manganese toxicity. *Sci. Rep.* **6**, 21113; doi: 10.1038/srep21113 (2016).

## Supplementary Material

Supplementary Information
