# Supplementary material for: Iron mitigates DMT1-mediated manganese cytotoxicity via the ASK1-JNK signaling axis: Implications of iron supplementation for manganese toxicity
Source: Sci Rep. 2016 Feb 16;6:21113. doi: 10.1038/srep21113 (PMC4754755; doi:10.1038/srep21113)

**Iron mitigates DMT1-mediated manganese cytotoxicity via the ASK1-JNK signaling axis: Implications of iron supplementation for manganese toxicity**

Yee Kit Tai, Katherine C. M. Chew, Bryce W. Q. Tan, Kah-Leong Lim, and Tuck Wah Soong

**Supplementary Figure Legends**

**Supplementary Figure 1. Full-length blots corresponding to Figure 3b.**

From top to bottom, blots were detected with anti-phospho-JNK, anti-JNK and anti-*β*-actin respectively. Lanes 1-3 correspond with the first three lanes of Figure 3b without JNK inhibitor, while lanes 7-9 correspond to the last three lanes of Figure 3b treated with JNK inhibitor SP600125.

**Supplementary Figure 2. Mn^2+^ mediates an increase in autophagy, while autophagy inhibition exacerbates Mn^2+^-mediated JNK activation.**

(a) Representative western blot of autophagy activation with Mn^2+^ treatment in S-DMT1 cells. 0.5 mM Mn^2+^ treatment for 12 h increased LC3-II protein levels (**p*<0.05) which was not observed with Fe^2+^ treatment. Co-treatment between Mn^2+^ and Fe^2+^ (both at 0.5 mM) resulted in a lower LC3-II protein level compared to Mn^2+^ treatment alone. *β*-actin was used as a control for equal loading. Bar chart shows the fold change of LC3-II protein levels over untreated (n=3, Student’s *t*-test), with error bars representing standard error of mean (S.E.M.). (b) Representative western analysis of Mn^2+^-mediated time-dependent JNK phosphorylation which is enhanced in the presence of lysosomal inhibition. Treatment of S-DMT1 cells with NH_4_Cl was for the duration of Mn^2+^ treatment as well as 4 h pre-treatment. Cells treated with 0.5 mM Mn^2+^ and NH_4_Cl exhibited heightened JNK phosphorylation compared to cells without NH_4_Cl (**p*<0.05, compared to no NH_4_Cl, n=4, Student’s *t*-test). Inhibition of lysosomal function was verified though the accumulation of autophagic LC3-II protein in the presence of NH_4_Cl.

**Supplementary Figure 3. Fe^2+^ repletion reduces Mn^2+^-mediated JNK phosphorylation and cell death.**

(a) Representative western analysis of JNK phosphorylation and ferritin levels upon Fe^2+^ repletion and Mn^2+^ treatment, together with corresponding full-length blots. Lanes 1 and 2 correspond in both sets, while lanes 3 and 4 correspond to lanes 5 and 6 of the full-length blots. Repletion with 0.5 mM Fe^2+^ for 12 h followed by 0.5 mM Mn^2+^ for 12 h resulted in a significant reduction in JNK phosphorylation and a corresponding increase in ferritin levels compared to Mn^2+^ treatment alone (**p*<0.05, n=3, Student’s *t*-test). (b) MTT assay showing increased S-DMT1 cell viability when co-treated with Fe^2+^ and Mn^2+^ for 24 h compared to Mn^2+^ treatment alone at 0.25 mM, 0.5 mM and 1 mM (***p*<0.01, ****p*<0.001, n=4, two-way ANOVA, Bonferroni post hoc test). Representative phase contrast images showing reduced vulnerability of S-DMT1 cells towards cytotoxic effect of 0.5 mM Mn^2+^ in the presence of 0.5 mM Fe^2+^.

Supplementary Figure 1


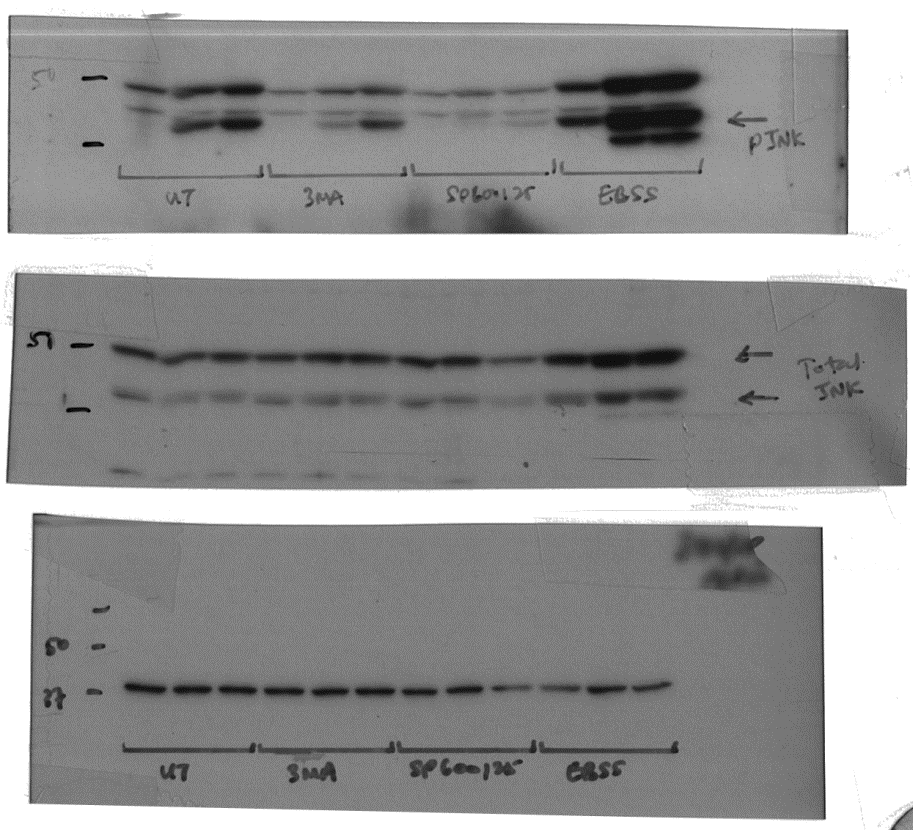


Supplementary Figure 2


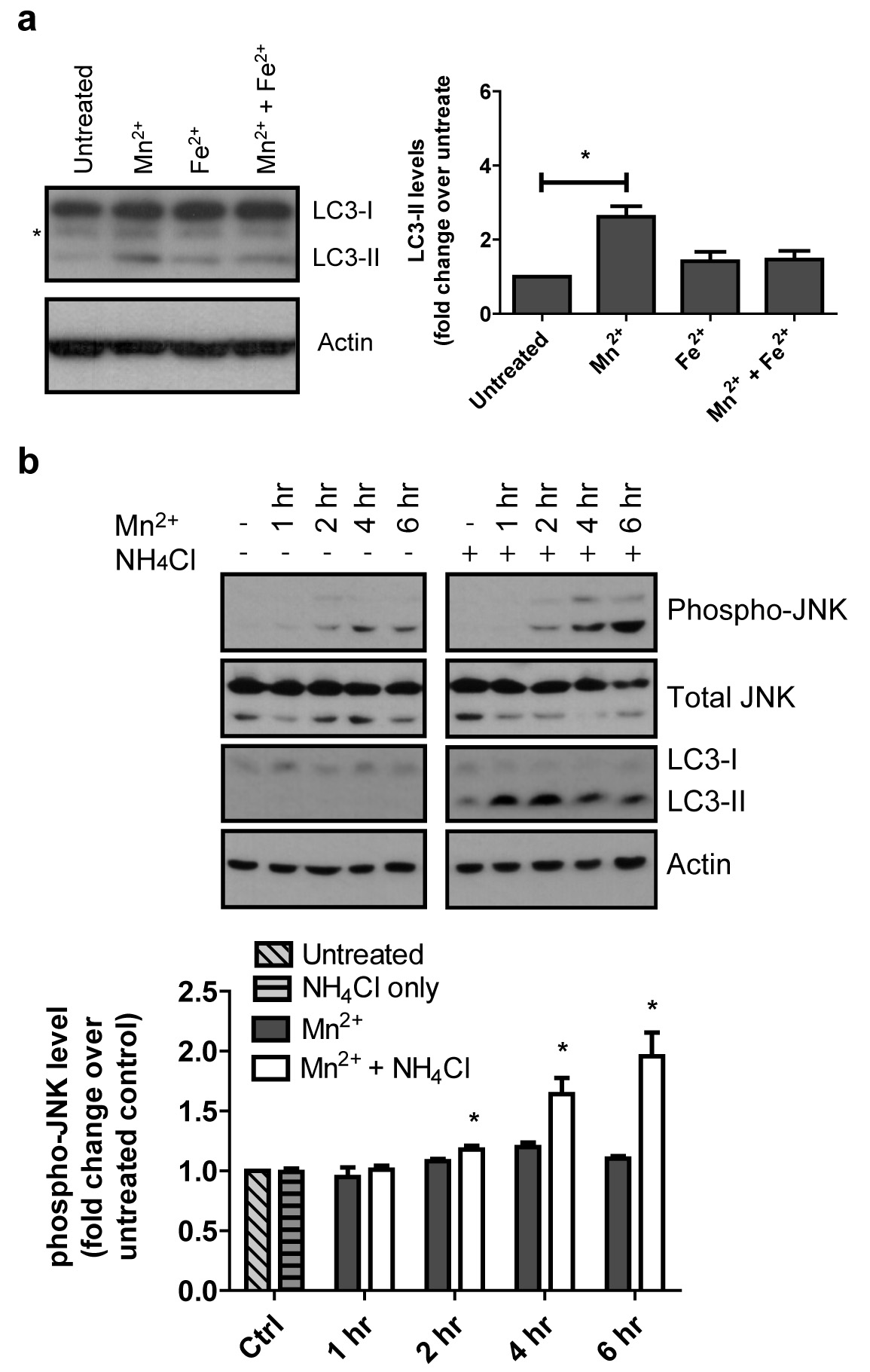


Supplementary Figure 3


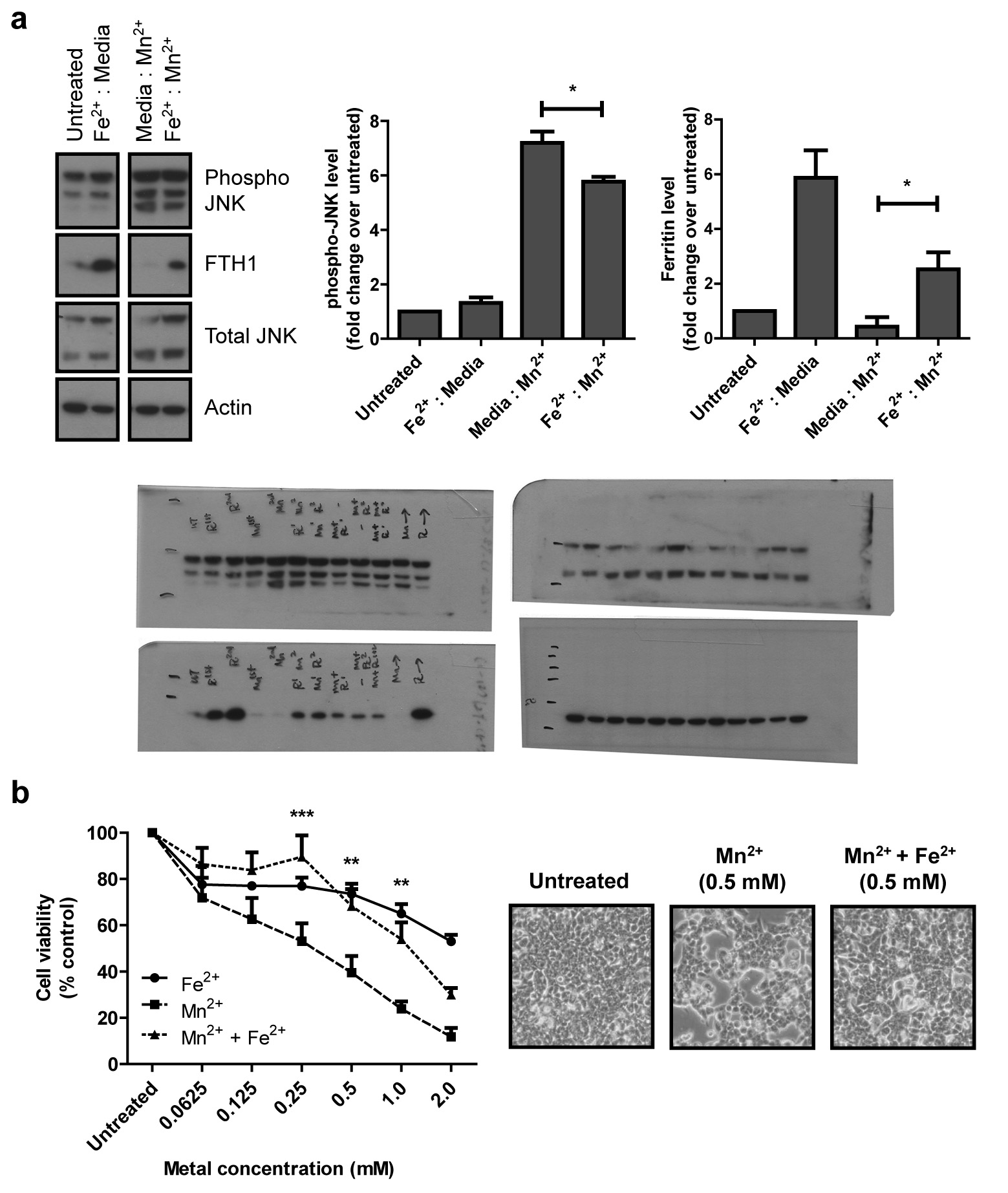

Supplement: Supplementary Information [file srep21113-s1.doc]
